# Application value of long-read sequencing in full characterization of thalassemia-associated structural variations: identifying a novel large segmental duplication and literature review

**DOI:** 10.1186/s13023-025-03701-8

**Published:** 2025-04-02

**Authors:** Zeyan Zhong, Ganwei Zheng, Dina Zhu, Yongqiong Liu, Zezhang Lin, Zhiyang Guan, Fu Xiong, Jianhong Chen, Xuan Shang

**Affiliations:** 1 Department of Medical Genetics and Prenatal Diagnosis, Huizhou First Maternal and Child Health Care Hospital, Huizhou, Guangdong China; 2 Department of Medical Genetics, School of Basic Medical Sciences, Southern Medical University, Guangzhou, Guangdong 510515 China; 3 Prenatal Diagnosis Center, Department of Obstetrics and Gynecology, Guangdong Provincial People’s Hospital (Guangdong Academy of Medical Sciences), Southern Medical University, Guangzhou, Guangdong China; 4 Reproductive Medicine Center, Jiangxi Maternal and Child Health Hospital Afliated to Nanchang Medical College, Nanchang, Jiangxi China; 5 Department of Fetal Medicine and Prenatal Diagnosis, Zhujiang Hospital, Southern Medical University, Guangzhou, China

**Keywords:** Thalassemia, Structural variation, Long-read sequencing

## Abstract

**Background:**

Thalassemia is one of the most prevalent monogenic disorders in tropical and subtropical regions, imposing significant familial and social burdens on local populations. It is caused by point mutations or structural variations (SVs) in the α- or β-globin gene clusters. Due to the complex structure, full characterization of SVs has always been the focus and difficulty of molecular diagnosis of thalassemia patients.

**Methods:**

Peripheral blood of a Chinese boy with β-thalassemia intermedia phenotype and his family members were collected. Multiplex ligation dependent probe amplification (MLPA), long-read sequencing (LRS) and Sanger sequencing were used to analyze the variant in this family.

**Results:**

A novel large duplication (αααα^280^) was identified using LRS technique and validated by Sanger sequencing. Additionally, we conducted a systematic review of known SVs and evaluated the advantages and disadvantages of various methods in analyzing complex SVs.

**Conclusions:**

Our study identified a novel SV in the α-globin gene cluster and demonstrated that LRS was a superior approach for detecting novel rare SVs. The appropriate use of LRS significantly improves diagnostic accuracy when conventional methods are not capable of completely identifying complex SVs.

**Supplementary Information:**

The online version contains supplementary material available at 10.1186/s13023-025-03701-8.

## Background

Thalassemia is one of the most common monogenic disorders globally, with high prevalence in tropical and subtropical regions such as Southeast Asia, the Mediterranean, the Indian subcontinent, the Middle East, and Africa [1]. It is estimated that approximately 5% of the global population are carriers of thalassemia variants, with at least 60,000 affected individuals born each year [2]. α-thalassemia Major, also known as Hb Bart’s hydrops fetalis, results in asphyxia and hydrops fetalis, often leading to in utero death. β-thalassemia Major causes severe anemia post-birth, requiring regular transfusions for survival, thus placing a significant burden on local health systems in endemic regions. A definite diagnosis of thalassemia genotype is the prerequisite in proper clinical management to thalassemia patients and affected families, so developing accurate and effective molecular diagnosis techniques has been a priority in thalassemia research.

Thalassemia is caused by variants in the α- and β-globin gene clusters [1]. According to statistical data from IthaGenes (https://www.ithanet.eu/db/ithagenes), more than 1,000 types of variants are associated with thalassemia in these two gene clusters. These variants can generally be categorized into three types: single nucleotide variations (SNVs), small insertions and deletions (indels), and structural variations (SVs). SNVs and indels refer to alterations shorter than 50 base pairs, while SVs are larger alterations spanning more than 50 base pairs, including large deletions (DELs), duplications (DUPs), inversions (INVs), insertions (INSs), translocations (TRAs), and more complex rearrangement events [3, 4]. SNVs and indels are easily detected by routine sequencing techniques, but full characterization of SVs poses a challenge to traditional methods due to their complex molecular structures.

Currently, gap polymerase chain reaction (Gap-PCR) could only be used to detect the most common SVs in thalassemia, such as Southeast Asian deletion (--^SEA^), 3.7 kb deletion (-α^3.7^), and 4.2 kb deletion (-α^4.2^). Multiplex ligation-dependent probe amplification (MLPA) and short-read sequencing (SRS), also known as next-generation sequencing (NGS), are able to detect the existence of rare SVs; but they usually difficult to discern the precise physical locations of breakpoints and structural characteristics of genomic rearrangements. In recent years, long-read sequencing (LRS) or third-generation sequencing (TGS) has emerged as a valuable tool for resolving the exact sequence composition of SVs, overcoming limitations related to assembly problems encountered with long and complex sequences [5, 6].In this study, we identified a novel large segmental duplication (αααα^280^) in a Chinese family by LRS method and then validated it by Sanger sequencing. We also summarized known large segmental duplication events in the α-globin gene cluster. Furthermore, we provide a comprehensive review of known SVs associated with thalassemia and evaluate the advantages and disadvantages of analyzing these complex SVs using MLPA, SRS, and LRS methods.

## Materials and methods

### Subjects

The proband was a boy from Jiangxi Province, southern China. He presented with moderate anemia and jaundice, and was diagnosed with β-thalassemia intermedia. Routine thalassemia mutation analysis in local hospital identified him as heterozygous for the β-thalassemia variant (*HBB*: c.316–197 C > T), which was inherited from his father. Family members were referred to our laboratory for further study with informed consent. The research protocol for this study was designed and implemented in accordance with the principles of the Declaration of Helsinki. Peripheral blood samples were collected. Hematological parameters were determined on an automated cell counter (Sysmex Co. Ltd., Kobe, Japan). Hemoglobin quantification was performed by capillary electrophoresis (Sebia, Montpellier, France).

### MLPA analysis

Multiplex ligation-dependent probe amplification (MLPA) assay was performed using the SALSA MLPA kit (MRC-Holland, the Netherlands) according to the instruction of the manufacturer. The MRC-Coffalyser software was used as analysis tool for the normalization of MLPA data [7].

### Long read sequencing

The Comprehensive Analysis of Thalassemia Alleles (CATSA) based on long read sequencing (LRS) was conducted as previously described [8]. Genomic DNA was subjected to multiplex PCR with primers covering the majority of known structural variations, SNVs and indels in the α- and β-globin gene clusters. A one-step end-repair and ligation reaction was then performed to add barcoded adaptors to the PCR products. Single-molecule real-time (SMRT) bell libraries were constructed using the Sequel Binding and Internal Ctrl Kit 3.0 (Pacific Biosciences, USA) and sequenced with Sequel II Sequencing Kit 2.0 under the circular consensus sequencing (CCS) mode. Long-read whole genome sequencing (LR-WGS) was also performed in Pacific Biosciences platform, too. Genomic DNA was extracted, qualified and quantified. Large-insert (15 kb) library was constructed using the SMRTbell Express Template Preparation Kit 2.0 (Pacific Biosciences, USA). After quality control test, the SMRTbell library was sequenced using a single 8 M SMAT Cell on the Sequel II system. Raw subreads were processed to CCS reads by CCS software (Pacific Biosciences), demultiplexed by barcodes using lima in the Pbbioconda package (Pacific Biosciences). Clean data was mapped to the reference human genome (hg38) by pbmm2 software. SNVs and indels were called by FreeBayes software. Structural variants were called from data using multiple tools.

### Gap-PCR and sanger sequencing

Gap-PCR and Sanger sequencing were used to precisely locate the exact breakpoint of this duplication. Primers were designed for the upstream and downstream duplication region as follows: F1 (5ʹ-GCACAAGGCCAGGAGAATGA − 3ʹ), R1(5ʹ-GGCTGTTCCAAAGTGTTGATGGC − 3ʹ), F2 (5ʹ-CCATCAGTGCCTAGTTTTAGCCAA − 3ʹ), R2 (5ʹ-GGAAGTGTCTTGCCTTGTGAAGGT − 3ʹ). PCR products were sequenced. Chromas software (Technelysium Pty Ltd., South Brisbane, Australia) was used to analyze the Sanger sequencing data.

## Results

### Clinical presentation

**Fig. 1:**
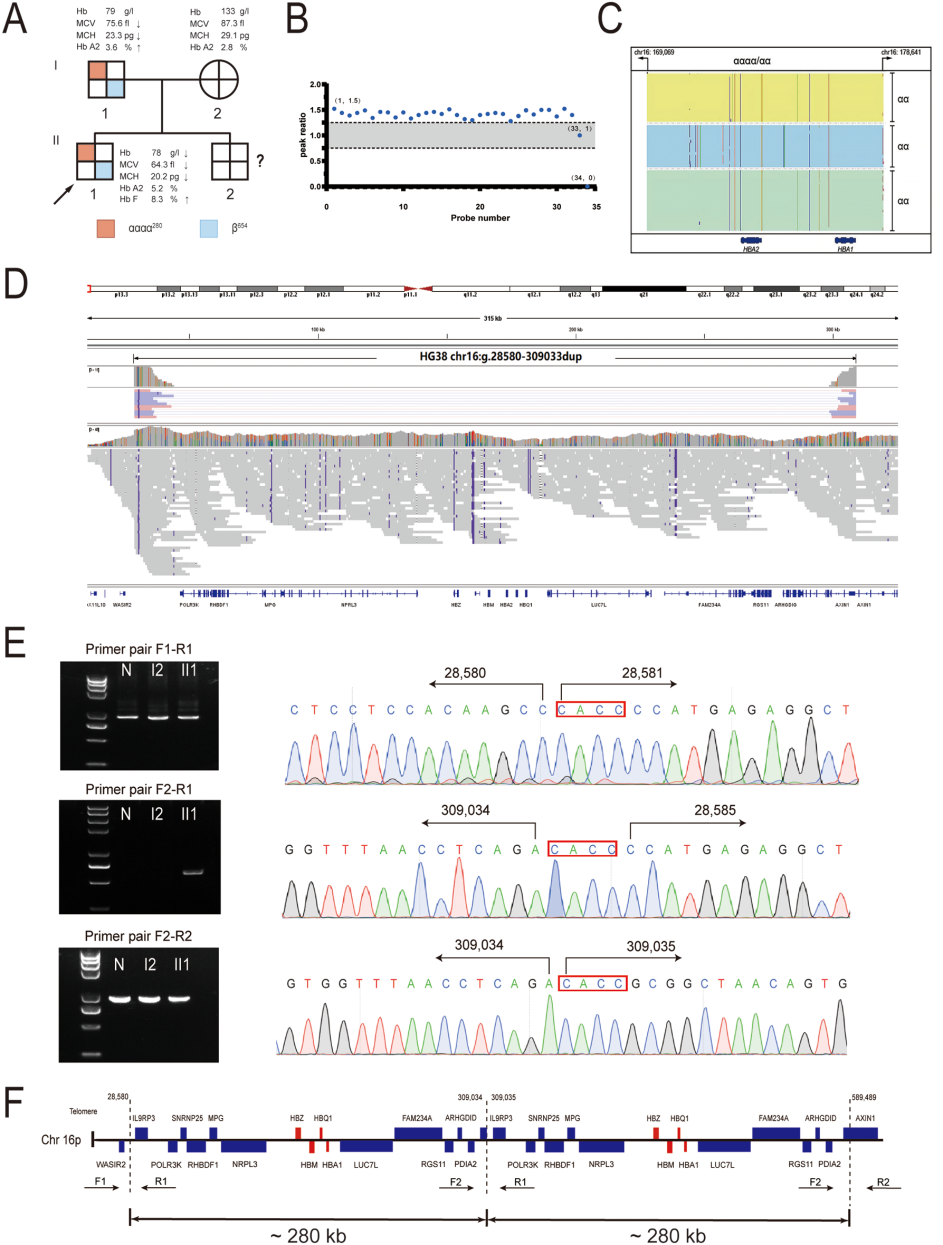
Identification of the novel αααα^280^ duplication (**A**) Family pedigree and phenotypic data. Sample of II2 was not obtained. (**B**) MLPA analysis of I1. The scatter plot shows the increase in copy number due to the duplicated region identified by MLPA analysis. Probe ratios between 0.7 and 1.3 were defined as normal and are indicated by gray areas. Probe 34 was designed for Constant Spring mutation detection. (**C**) Integrative Genomics Viewer plots for I1 by CATSA assay showed three αα haplotypes with three different colored area. (**D**) LR-WGS method identifies a complex variant: chr16:g.28580-359033dup (280,453 bp). (**E**) Identification of breakpoints of the duplication. Three pairs of primers F1-R1, F2-R1, F2-R2 were used for PCR analysis. The breakpoint of the duplication was amplified by primers F2 and R1, where a unique 588 bp product was amplified in II1, but not in I2 or the normal individual (N). Direct sequencing results of the products showed that the duplication region started between positions 28,580 and 28,585 and ended between 309,034 and 309,039. Sequences 309,035–309,038 and 28,581 − 28,584 (red boxes) are identical. (**F**) Schematic representation of the duplication structure

The proband II1 (Fig. 1A), a young boy, presented with moderate anemia (Hb 78 g/L) and was preliminarily diagnosed with β-thalassemia intermedia based on blood testing, which confirmed microcytic (MCV 64.3 fL, RI: 80–100 fL) and hypochromic characteristics (MCH 20.2 pg, RI: 27–34 pg), elevated Hb A_2_ level (5.2%, RI: 2.5–3.5%) and Hb F level (8.3%, RI: 0–2%). Iron deficiency was excluded. Conventional analysis of common thalassemia mutations in the Chinese population at the local hospital detected only a paternal inheritance of a heterozygous β-thalassemia mutation (β^654^, *HBB*: c.316–197 C > T). However, this heterozygous β-thalassemia mutation alone was insufficient to explain the β-thalassemia intermedia phenotype, prompting further investigations into other variants relevant to imbalanced production of globin chains. Family members were referred to our laboratory for further study with informed consent. The study was designed and implemented in accordance with the principles of the Declaration of Helsinki. Pedigree analysis revealed that the father exhibited a similar anemia phenotype and had a history of receiving transfusions in childhood, suggesting that the unknown variant should also be inherited from the father.

MLPA analysis (Fig. 1B) detected a large duplication that included the α-globin genes, but the exact range could not be determined. To identify this structural variation, a reported method (CATSA) [8] based on LRS technology (Pacific Bioscience platform) was performed as previously described [9]. However, this targeted LRS assay also failed to identify the exact structure of this gene rearrangement. Haplotype analysis displayed three haplotypes of complete α2 and α1 genes, indicating that there were four α-globin genes on one allele and two α-globin genes on the other allele (Fig. 1C). This confirmed the presence of a large segmental duplication in this family and suggested that the duplication was too large, exceeding the detection scope of the CATSA assay. Therefore, we used long-read whole genome sequencing (LR-WGS) (Pacific Bioscience platform) to elucidate the detailed structure of this duplication. LR-WGS analysis revealed a duplication located at hg38/chr16: 28,580–309,033 (Fig. 1D). This duplication encompassed a contiguous segment of approximately 280 kb and occurred in a tandem arrangement. Based on this precise range estimation, two pairs of primers were designed. PCR products of 887 and 905 bp were obtained using primers F1-R1 and F2-R2, respectively (Fig. 1E). A unique Gap-PCR product of 588 bp was generated from the proband (II1) using primers F2-R1. Sanger sequencing of this product validated a 280,454 bp tandem duplication fragment between positions 28,580 − 28,585 and 309,034–309,039. Sequences in the 28,581 − 28,585 and 309,035–309,039 regions all shared a CACC read (Fig. 1E). Interestingly, this was almost identical to the break location and length expected by the LR-WGS analysis.

This is a novel large segmental duplication (αααα^280^) that has not been previously reported. Therefore, the proband (II1) and his father (I2) possessed compound heterozygote variants comprising a known β^654^ mutation and a large duplication of αααα^280^. This duplication increased the number of α-globin genes from four to six, exacerbating the imbalance between the α-globin and β-globin chain ratio, and leading to the phenotype of β-thalassemia intermedia.

**Fig. 2:**
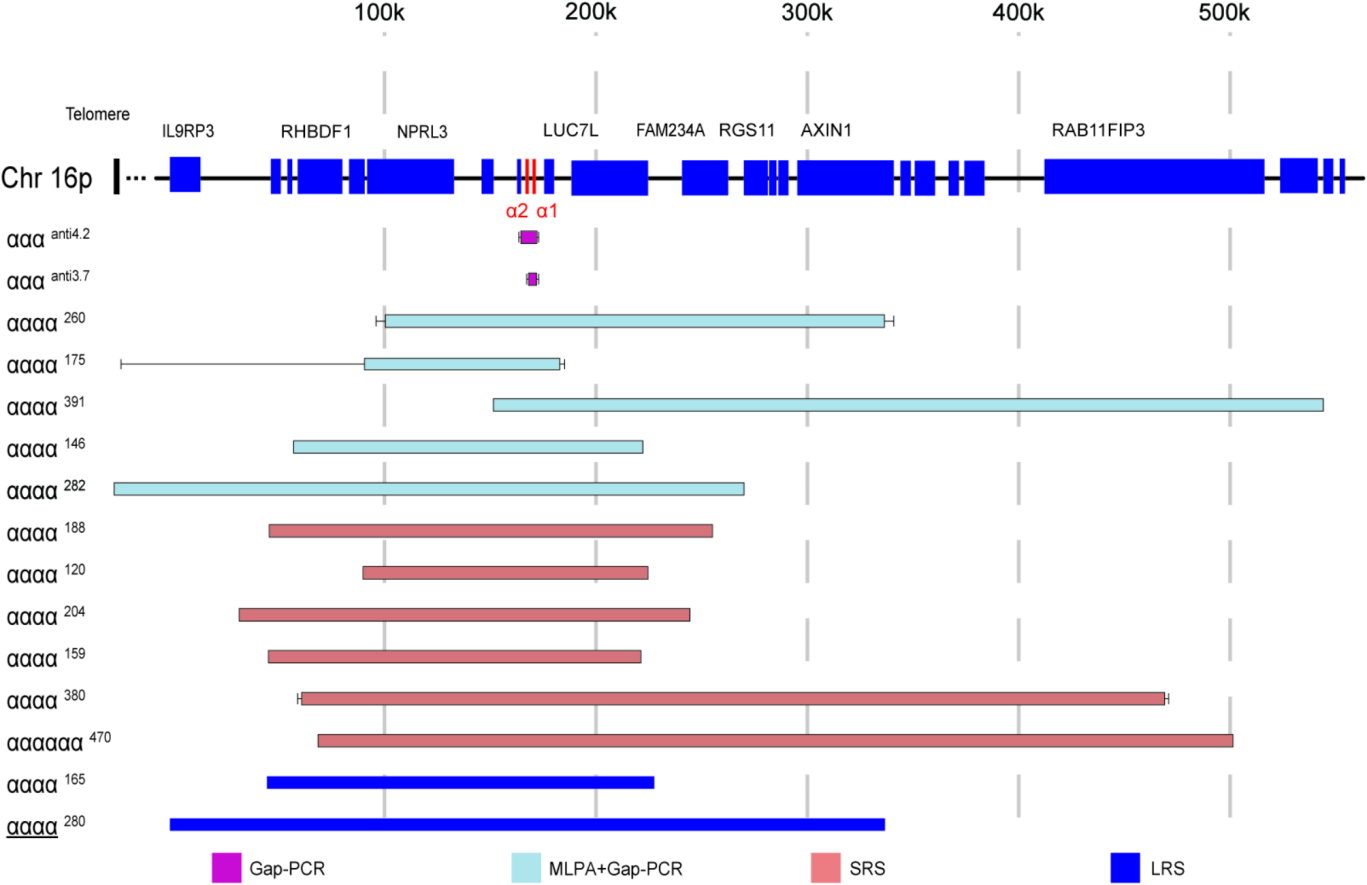
Overview of the known duplications involving the α-globin gene with precise breakpoints. Fifteen duplications are shown. The uncertainties of the five special breakpoints (ααα^anti4.2^, ααα^anti3.7^, αααα^260^, αααα^175^and αααα^380^) are represented by black lines. The novel duplication reported in this paper (αααα^280^) is underlined in the text. αααααα^470^ has a complex structure as follows: GRCh38 dup(16)(p13.3), trp(16) (p13.3),dup(16)(p13.3) NC_000016.9:g.pter_502829;67268_545444.502816_503125inv;63444_63603inv;83416_qter. Only duplicated region was shown, the inversion region is not shown. Genes in this region are represented by blue bars, while the two α-globin genes (α2 and α1)are represented by red bars. Identification methods for these duplications are indicated by various colors.

In addition, we summarized previously reported duplication events involving the α-globin gene. A total of 15 duplications [7, 10–22]. with detailed molecular structure data was reviewed (Fig. 2A) Among them, ααα^anti3.7^ and ααα^anti4.2^ are prevalent in thalassemia-endemic regions in southern China [23, 24], while other duplications are rare SVs and have only been detected in sporadic cases. The breakpoint information of these rare duplications is shown in Table S1 (in supplement material), and phenotype data of individuals carrying these duplications or compound heterozygous for β-thalassemia and these duplications were summarized in Table S2 (in supplement material). All of these rare duplications occur in tandem and are in direct orientation to the original locus. With the development of sequencing technology, six duplications (αααα^188^, αααα^120^, αααα^204^, αααα^159^ αααα^380^ and αααααα^470^) reported earlier were identified using the SRS technique, while recently discovered two duplications (αααα^165^ and αααα^280^) were characterized using the LRS technique.

## Discussion

Structural variations (SVs) in the α- and β-globin gene clusters are important disease-causing or genetic modifier factors of thalassemia [1]. For example, the --^SEA^ deletion, -α^3.7^ deletion, -α^4.2^ deletion, and the 619 bp deletion in the β-globin gene are major causes of α-thalassemia in southern China and β-thalassemia in India, respectively [25]. Heterozygotes for β-thalassemia mutations co-inherited with duplications in the α-cluster, such as α-triplication (αα/ααα^anti3.7^ or αα/ααα^anti4.2^), display mild to moderate anemia phenotypes rather than asymptomatic thalassemia trait phenotypes [20]. Therefore, accurate identification of SVs is crucial for the precise diagnosis of thalassemia.

**Fig. 3:**
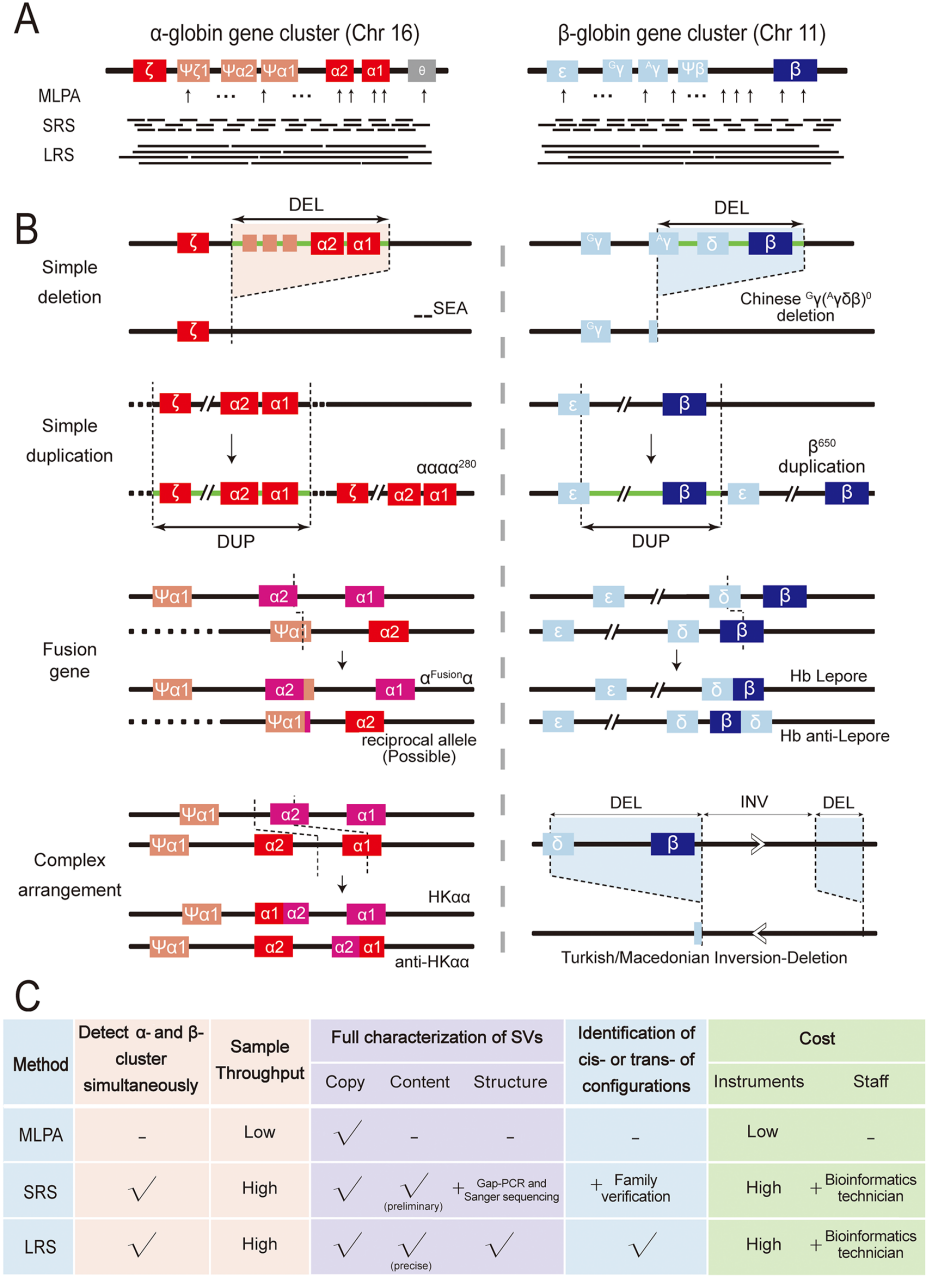
Evaluate the advantages and disadvantages of analyzing thalassemia-associated SVs using MLPA, SRS, and LRS methods (**A**) The structure of the α- and β-globin gene clusters. (**B**) Schematic diagram showing the structure of four types of SVs. DUP for duplication, DEL for deletion, and INV for inversion. (**C**) Comparison of detecting SVs using MLPA, SRS and LRS methods

The structure of the α- and β-globin gene clusters is shown in Fig. 3A. The α-cluster contains an embryonic gene (ζ, HBZ), two fetal/adult α genes (α2, HBA2. α1, HBA1), two pseudogenes (ψζ1, and ψα1,), and two minor globin-like genes (ψα2, HBM. θ, HBQ1), arranged in the following order: ζ-ψζ1-ψα2-ψα1-α2-α1-θ. The α1 and α2 are nearly identical genes. The β-cluster contains an embryonic gene (ε, HBE), two fetal genes (Gγ, HBG2. Aγ, HBG1), one pseudogene (ψβ), and two adult genes (δ, HBD. β, HBB), arranged in the following order: ε- Gγ - Aγ-ψβ-δ-β. Genes in the α- and β-clusters exhibit a high degree of homology, with recombination events between homologous sequences within the clusters occurring readily [14].

To date, hundreds of structural variations (SVs) in the α- or β-globin gene clusters have been identified. We systematically reviewed known SVs and classified them into four types according to their structural characteristics (Fig. 3B). “Simple deletion” or “Simple duplication” refers to those SVs in which a contiguous fragment containing key functional genes (such as α2, α1, and β) or major regulatory elements (such as HS-40 in the α-globin cluster) was deleted or duplicated [1]. These alterations change the copy number of globin genes or important globin expression regulatory elements, resulting in increased or decreased levels of globin synthesis, ultimately causing thalassemia. For instance, the --^SEA^ deletion leads to the loss of two α genes; the Chinese ^G^γ(^A^γδβ)^0^ deletion removes three globin genes (^A^γ,δ and β); the αααα^280^ duplication(our study) results in α-quadruplication; and the 650 kb duplication leads to two copies of the β gene [1, 26]. “Fusion gene” refers to a hybrid gene formed by the crossover of two globin genes, typically generated by recombination events during meiosis. An α^Fusion^α variant is a fusion between the α2 and ψα1 genes, which alters the 3’UTR of the α2 gene and affects the polyadenylation signal, leading to α^+^-thalassemia [27]. Hb Lepore is a product of the δβ fusion gene. “Complex rearrangement” refers to SVs characterized by at least two crossover or recombination events. For example, the HKαα allele is a rearrangement containing both the -α^3.7^ and ααα^anti4.2^ unequal crossover junctions, whereas the anti-HKαα allele is the reciprocal product containing both the -α^4.2^ and ααα^anti3.7^ unequal crossover junctions [28]. The Turkish/Macedonian inversion-deletion allele contains three characteristic regions: two deletions and one inversion [26]. Taken together, while the majority of thalassemia-associated SVs present with relatively simple structures, recent research has identified some “complex” SVs. These complex SVs exhibit intricate rearrangements or involve more subtle changes. Due to the complexity and multifaceted nature of SVs, their full characterization presents a significant challenge for current molecular testing technologies.

Currently, commonly used techniques for detecting thalassemia-associated structural variations (SVs) include Gap-PCR, MLPA, SRS, and LRS. Gap-PCR is the most commonly used method in clinical laboratories, but it can only detect a limited number of common deletions. In contrast, MLPA, SRS, and LRS can detect rare SVs and discover new SVs. However, these techniques differ in their capacity for comprehensive assessment of SVs. We compare them in Fig. 3C.

First, two benefits of SRS and LRS over MLPA are their ability to simultaneously detect α- and β-globin clusters and their high sample throughput. In contrast, MLPA needs two steps because of two sets of probes corresponding to α- and β- cluster separately so as to cumbersome and labor-intensive. Second, for the full characterization of SVs, three features need to be accurately described: copy (the copy number of important globin genes), content (the range of the SV), and structure (breakpoints, insertion direction, tandem or interspersed duplication, etc.). Due to the limited number of probes and their focus on the α- and β-globin clusters, MLPA can detect globin gene copy number alterations caused by deletions or duplications. However, it struggles to estimate the range of large deletions or duplications that extend beyond clusters. Sequencing reads from SRS are typically short (< 500 bp). With the conditions of high coverage of genome and high sequence depth, SRS can determine globin gene copy numbers and make a preliminary estimation of the range of SVs. Further identification of breakpoints requires Gap-PCR and Sanger sequencing. This combination of techniques has been successfully applied in discovering rare SVs, such as the duplications listed in Fig. 2. However, due to the highly homologous nature of α2 and α1 genes and the GC-rich regions in the α-globin cluster, detecting complex rearrangements is challenging for SRS because of the technology’s natural limitations such as short read lengths and GC-content bias. LRS overcomes these limitations by producing continuous reads (> 10 kb) that span longer genomic stretches (across exons, genes, pseudogenes, and highly repeated regions), enabling more precise detection of SVs. It is estimated that LRS provides a fivefold increase in sensitivity for SV detection compared with SRS [29]. The nature of long reads permits accurate haplotype phasing, which is superior for determining long-range structures and precise breakpoints simultaneously. Notably, our results showed that breakpoints detected by LRS are at base pair resolution. Unlike SRS, which requires additional confirmation through Gap-PCR and Sanger sequencing, full characterization of thalassemia-associated SVs can be completed by LRS in a “one-step” manner. Moreover, LRS allows for the distinction of whether two or more variants are in cis- or trans-configurations simultaneously, while SRS requires additional family verification. This advantage indicates a shorter laboratory turnaround time for LRS, making it more suitable for clinical scenarios with high timeframe requirements, such as prenatal diagnosis.

The technological advantages of LRS are clear, but several disadvantages restrict its broad usage in clinical practice. The major limitation is the high cost of instruments (sequencing equipment and computational infrastructure for data analysis) and reagents (library preparation and sequencing), which is much higher than that of MLPA and relatively higher than that of SRS. Additionally, both SRS and LRS require the support of skilled bioinformatics technicians and optimized analytical algorithms. Therefore, in clinical laboratories, especially start-up laboratories, researchers need to find a balance between cost and performance. They should decide on strategies based on their overall goals and budget.

LRS is more ideal for resolving complex SVs as well as SVs in repetitive regions. It has been applied in the diagnosis of many high-prevalence genetic diseases, including thalassemia, congenital adrenal hyperplasia, hemophilia A, Duchenne muscular dystrophy, spinal muscular atrophy, and fragile X syndrome [5, 30]. It has also shown excellent performance in detecting cancer-related SVs. LRS increases the diagnostic rate and reduces the time to achieve a definitive diagnosis. When the high-cost issue is addressed through the development of hardware and software systems, we are optimistic about seeing a wider application of LRS in more clinical practices.

## Electronic supplementary material

Below is the link to the electronic supplementary material.

Supplementary Material 1

## Data Availability

Not applicable.
