# Supplementary material for: Application value of long-read sequencing in full characterization of thalassemia-associated structural variations: identifying a novel large segmental duplication and literature review
Source: Orphanet J Rare Dis. 2025 Apr 2;20:153. doi: 10.1186/s13023-025-03701-8 (PMC11963660; doi:10.1186/s13023-025-03701-8)
Supplement: Supplementary file 1 — Supplementary Material 1 [file 13023_2025_3701_MOESM1_ESM.docx]

Supplementary Table

**Table S1** Summary of duplications involving α-globin gene

| Case | Size | Telomeric breakpoint | Centromeric breakpoint | Genes involved  （numbers） | Reference |
| --- | --- | --- | --- | --- | --- |
|  | (kb) |  |  |  | (PMID) |
| ααα^anti4.2^ | 4.2 | Inside blocks X2 | Inside blocks X1 | HBA2, HBA1  (2) | 7440717 |
| ααα^anti3.7^ | 3.8 | Inside blocks Z2 | Inside blocks Z1 | HBA2, HBA1  (2) | 6928643 |
| αααα^391^ | 391 | 151,483 | 542,029 | HBZ…HBA2, HBA1…RAB11FIP3  (18) | 25690803 |
| αααα^146^ | 146 | 85,880 | 232,097 | NPRL3…HBA2, HBA1…LUC7L  (10) | 26086873 |
| αααα^282^ | 282.1 | Telomere | 282,199 | DDX11L10…HBA2, HBA1…ARHGDIG  (17) | 27000657 |
| αααα^188^ | 188.8 | 62,166 | 250,992 | RHBDF1…HBA2, HBA1…FAM234A  (13) | 27469621 |
| αααα^120^ | 120.5 | 98,543-98,546 | 219,039-219,042 | NPRL3…HBA2, HBA1…LUC7L  (10) | 27469621 |
| αααα^204^ | 203.9 | 47,553-47,555 | 251,522-251,524 | POLR3K…HBA2, HBA1…FAM234A  (10) | 31124576 |
| αααα^159^ | 159.4 | 63,142-63,147 | 222,524-222,529 | RHBDF1…HBA2, HBA1…LUC7L  (14) | 36326959 |
| αααα^380^ | 380 | 80,000±20 | 460,000±20 | MPG…HBA2, HBA1…RAB11FIP3  (20) | 37194740 |
| αααα^165^ | 165 | 59,867 | 224,557 | RHBDF1…HBA2, HBA1…LUC7L  (12) | 38693050 |
| αααααα | - | 83,416 | 502,829 | MPG…HBA2, HBA1…RAB11FIP3  (20) | 38817045 |
| αααα^260^ | 260 | The α2-globin gene to 75 kb upstream | The α2-globin gene to 185 kb downstream | MPG…HBA2, HBA1…AXIN1  (15) | 18249014 |
| αααα^175^ | 175 | Telomere | The 175 kb region of the 3′ HVR | MPG…HBA2, HBA1  (9) | 18249014 |
| αααα^280^ | 280 | 28,580 | 359,033 | IL9RP3…HBA2, HBA1…AXIN1  (17) | Our study |

Coordinates refer to the chromosome 16 assembly of the reference genome (GRCh38/hg38, December 2013)

αααα^282^ is an insertion duplication.

αααααα is a complex structure as follows: seq[GRCh37] dup(16)(p13.3),trp(16) (p13.3),dup(16)(p13.3) NC_000016.9: g.pter_552829;117266_595444.552816_553125inv;113442_113601inv;133415_qter. Convert position to GRCh38 dup(16)(p13.3),trp(16) (p13.3),dup(16)(p13.3) NC_000016.9:g.pter_502829;67268_545444.502816_503125inv;63444_63603inv; 83416_qter.

**Table S2** Summary of the hematological data of individuals carrying duplications involving α-globin gene

| **Smaple** | **Gender** | **α-globin**  **genotype** | **β-globin**  **genotype** | **MCV (fL)** | **MCH (pg)** | **Hb (g/dL)** | **Hb A₂ (%)** | **Hb F (%)** |
| --- | --- | --- | --- | --- | --- | --- | --- | --- |
|  | **/Age** |  |  |  |  |  |  |  |
| **αααα^391^** |  |  |  |  |  |  |  |  |
| A1 | M/- | αααα^391^/αα | β/β | 90.1 | 30.2 | 15.6 | 2.8 | 0.7 |
| A2 | F/7 | αααα^391^/αα | β^IVSII-654^/β | - | - | 5.6 | 2.8 | 2.4 |
| **αααα^146^** |  |  |  |  |  |  |  |  |
| B1 | M/29 | αααα^146^/αα | β/β | 77.4 | 26.5 | 12.7 | 2.7 | 0.9 |
| B2 | M/4 | αααα^146^/αα | β^IVSII-654^/β | 69 | 19.4 | 5.5 | 5.3 | 6.1 |
| **αααα^282^** |  |  |  |  |  |  |  |  |
| C1 | M/40 | αααα^282^/αα | β/β | 77.4 | 26.1 | 14.3 | 2.6 | 0.6 |
| C2 | M/7 | αααα^282^/αα | β^CD41-42^/β | 63.7 | 18.3 | 6.1 | 4.5 | 7.7 |
| **αααα^188^** |  |  |  |  |  |  |  |  |
| D1 | M/54 | αααα^188^/αα | β^IVSII-654^/β | 63.6 | 19 | 6.7 | 3.7 | 2.6 |
| D2 | M/14 | αααα^188^/αα | β^IVSII-654^/β | 68.2 | 21.1 | 9.1 | 4.2 | 9.5 |
| **αααα^120^** |  |  |  |  |  |  |  |  |
| E1 | M/- | αααα^120^/αα | β/β | 80.1 | 28.2 | 15.9 | 2.7 | 0.2 |
| E2 | F/- | αααα^120^/αα | β^CD44-C^/β | 65.6 | 19.6 | 5.9 | 3.1 | 13.6 |
| E3 | F/- | αααα^120^/αα | β^CD44-C^/β | 68.9 | 20.7 | 8.5 | 4.2 | 15.2 |
| **αααα^204^** |  |  |  |  |  |  |  |  |
| F1 | M/- | αααα^204^/αα | β^IVSII-654^/β | 74.6 | 22.7 | 6.7 | 3.4 | 12.5 |
| **αααα^159^** |  |  |  |  |  |  |  |  |
| G1 | F/26 | αααα^159^/αα | β/β | 81.9 | 27.2 | 11.4 | 3.3 | 0.6 |
| G2 | F/4 | αααα^159^/αα | β^CD41-42^/β | 60.5 | 17.2 | 7.5 | 4.9 | 11.3 |
| **αααα^380^** |  |  |  |  |  |  |  |  |
| H1 | F/42 | αααα^380^/αα | β/β | 77.9 | 25.5 | 12.1 | 2.5 | 0 |
| H2 | M/14 | αααα^380^/αα | β/β | 70.9 | 23.1 | 14.2 | 2.7 | 0 |
| H3 | M/10 | αααα^380^/αα | β^CD41-42^/β | 68 | 19.1 | 6.4 | 3.9 | 8.4 |
| **αααα^165^** |  |  |  |  |  |  |  |  |
| I1 | M/ | αααα^165^/αα | β/β | 83.5 | 28.2 | 14.8 | 2.8 | 0 |
| I2 | F/ | --^SEA^/αα | β^CD17^/β | 67.1 | 21.7 | 11.6 | 5.8 | 0 |
| I3 | M/6 | αααα^165^/αα | β^CD17^/β^c.-175(G>A)^ | 62.4 | 17.9 | 8.3 | 4.2 | 3.5 |
| **αααααα** |  |  |  |  |  |  |  |  |
| J1 | 50/f | αααααα/αα | β/β | 85.7 | 28.6 | 14.2 | 2.9 | 0.6 |
| J2 | 19/m | αααααα/αα | β/β | 75 | 24.8 | 13.3 | 2.7 | 0.7 |
| J3 | 15/f | αααααα/αα | β^AATAAG^/β | 60.8 | 17.9 | 9 | 3 | 0.8 |
| J4 | 10/m | αααααα/αα | β^AATAAG^/β | 57.3 | 16.8 | 7.7 | 2.9 | 1 |
| **αααα^260^** |  |  |  |  |  |  |  |  |
| K1 | F/30 | αααα^260^/αα | β^CD 39^/β | 68.9 | 24.1 | 8 | 3.8 | 17.6 |
| **αααα^175^** |  |  |  |  |  |  |  |  |
| L1 | F/54 | αααα^175^/ααα^anti3.7^ | β^CD 39^/β | 66.1 | 25 | 8 | - | - |
| **αααα^280^** |  |  |  |  |  |  |  |  |
| M1 | M/34 | αααα^280^/αα | β^IVSII-654^ /β | 76.6 | 23.3 | 79 | 3.6 | - |
| M2 | M/13 | αααα^280^/αα | β^IVSII-654^ /β | 64.3 | 20.2 | 78 | 5.2 | 8.3 |

These data were collected from the literature listed in Table S1. Transfusion data was not listed in the table due to these data rarely reported in the literature.

MCV: mean corpuscular volume; MCH: mean corpuscular hemoglobin. Hb: hemoglobin.

HGVS Name of the β-globin mutations: β^CD39^, HBB:c.118C>T. β^AATAAG^, HBB:c.*113A>G. β^IVSII-654^, HBB:c.316-197C>T. β^CD41-42^ , HBB:c.126_129delCTTT. β^CD 44-C^, HBB:c.135delC. β^CD17^, HBB:c.52A>T. β^c.-175(G>A^ ,HBB:c.−175G > A.
